# MEETINGS CALENDAR 2016

**Published:** 2016

## July

**1 to 3 - Aortic Valve Repair Summit 2016**
Brussels, Belgium
Informations:
Phone: +3225378701
E-mail: registration@co-mana.com
Site: valvesymposium.com

**7 to 9 - 5^th^ Annual Chicago CardiovascularUpdate**
Chicago, United States
Informations: Office of Continuing Medical Education
Phone: 312-503-8533
Fax: 312-503-4531
E-mail: cme@northwestern.edu
Site: www.cme.northwestern.edu

**11 and 12 - Annual Conference on Atherosclerosis**
Philadelphia, United States
Informations: Eva Jones
Phone: 1-702-508-5200: 8033
E-mail: atherosclerosis@insightconferences.com
Site: http://atherosclerosis.conferenceseries.com

**14 to 16 - 4^th^ Singapore VALVE 2016**
Singapore, Singapore
Informations: Course Secretariat
E-mail: nhccme@nhcs.com.sg

**23 to 26 - Heart Valve-Related Disorders Conference**
Cambridge, United Kingdom
Informations: Matthew Kirkby
Phone: 01223750020
E-mail: matthew.kirkby@zingconferences.com

## August

**11 and 12 - International Conference on Hypertension & Health Care**
Toronto, Canada
Informations: Jessie Alison
Phone: 7025089022
E-mail: hypertension@conferenceseries.com
Site: http://hypertension.conferenceseries.com/

**11 and 12 - AATS Cardiovascular Valve Symposium**
Beijing, China
Informations: Meetings at AATS
Phone: 978-927-8330
E-mail: meetings@aats.org
Site: http://aats.org/valvebeijing/

## September

**2 and 3 - Endocarditis Symposium**
Seoul, South Korea
Informations: Pyowon Park
E-mail: pwpark@skku.edu
Site: http://www.valveforum.org

**3 to 6 - EACTA Echo Course 2016**
Istanbul, Turkey
Informations: Randi Wilson
E-mail: eacta@ics.dk
Site: http://www.eacta.org/echocourses/eacta-echo-course-2016

**8 to 11 - 26^th^ World Society of Cardiothoracic Surgeons 2016 Congress Combined With South African Heart Association Annual Meeting 2016**
Cape Town, South Africa
Informations: Helene Uys
Phone: +27 31 303 9852
Fax: +27 31 303 9529
E-mail: info@wscts2016.co.za
Site: www.wscts2016.co.za/

**9 and 10 - 2^nd^ North American Aortic Valve Repair Symposium**
Philadelphia, United States
Informations: Ansheia Spence
Phone: 215-898-6400
E-mail: penncme@mail.med.upenn.edu

**10 - Pan-African Society for Cardiothoracic Surgery (PASCaTS) Forum 2016 in Conjunction With the WSCTS Meeting**
Cape Town, South Africa
Informations: Prof. Charles Yankah and Dr. Willie Koen
Phone: +49-172-3020143
E-mail: cyankah@web.de, wkoen@iafrica.com
Site: www.pascats.com

**13 and 14 - 3^rd^ International Surgical Aspects of Cardiopulmonary Transplantation Course**
Newcastle upon Tyne, United Kingdom
Informations: Anna Burley
E-mail: anna.burley@aesculap-academy.com
Site: www.aesculap-academia.co.uk

**14 to 16 - Reconstruction of the Aortic Valve and Root: A Practical Approach**
Homburg, Germany
Informations: Jessica Kuenstler
Phone: +4961829466636
Fax: +4961829466644
E-mail: j.kuenstler@kelcon.de

**15 to 17 - 2016 Duke Masters of Minimally Invasive Thoracic Surgery**
Orlando, United States
Informations: Patti Deshaies
Phone: 919-681-6370
E-mail: patricia.deshaies@duke.edu

**15 to 17 - 13^th^ Annual Conference CVT Critical Care 2016**
Washington, United States
Informations: Mowahib Vermillion
Phone: 703-992-9948
E-mail: info@facts-care.org
Site: http://www.facts-care.org/

**15 to 17 - Joint Conference on Advances in Pediatric Cardiovascular Disease Management**
Anaheim, United States
Informations: Martha Gomez
Phone: (323) 361-4941
Fax: (323) 361-3668
E-mail: mgomez@chla.usc.edu
Site: www.chla.org/event/cme-events

**17 to 19 - Echo Northwestern 2016 38^th^ Annual Program**
Chicago, United States
Informations: Office of Continuing Medical Education
Phone: 312-503-8533
Fax: 312-503-4531
E-mail: cme@northwestern.edu

**21 and 22 - Surgical Morphology and Imaging of Congenital Heart Disease**
Singapore, Singapore
Informations: Secretariat, the Academia
E-mail: sims.sssc@singhealth.com.sg
Site: http://www.singhealthacademy.edu.sg/sdc2016

**22 to 24 - Birmingham Review Course in Cardiothoracic Surgery**
Birmingham, United Kingdom
Informations: Lorraine Richardson
Phone: +447711132946
Fax: 01296 733 823
E-mail: lorrainerichardson1@btinternet.com
Site: www.birminghamreviewcourse.co.uk

**23 and 24 - 3^rd^ Symposium on Coronary Artery Anomalies**
Philadelphia, United States
Informations: Ms. Micah Holliday
Phone: 215-590-5263
Fax: 215-590-4342
E-mail: hollidaydm@email.chop.edu
Site: www.chop.edu/aaoca-2016

**23 and 24 - Lung Cancer Alliance Sceening and Care Conference**
Washington, United States
Informations: Amy Copeland
E-mail: screening@lungcanceralliance.org

**28 to 30 - Advances in Quality & Outcomes: A Data Managers Meeting**
Baltimore, United States
Informations: Laura Medek
Phone: (312) 202-5839
E-mail: lmedek@sts.org

**30 to 01- 3rd Annual Chicago CSI (Case-Based Coronary and Structural Heart Intervention) Update**
Chicago, United States
Informations: Office of Continuing Medical Education
Phone: 312-503-8533
Fax: 312-503-4531
E-mail: cme@northwestern.edu

